# Absolute Weight Loss, and Not Weight Loss Rate, Is Associated with Better Improvements in Metabolic Health

**DOI:** 10.1155/2019/3609642

**Published:** 2019-01-29

**Authors:** Jennifer L. Kuk, Rebecca A. G. Christensen, Sean Wharton

**Affiliations:** ^1^School of Kinesiology and Health Science, York University, Toronto, Canada; ^2^The Wharton Medical Clinic, Hamilton, Canada

## Abstract

**Objective:**

To determine if the rate of weight loss (WL) is associated with metabolic changes independent of the absolute WL.

**Methods:**

WL and health changes were assessed in 11,281 patients attending a publicly funded clinical weight management program over a treatment period of 12.7 months. Early weight loss rate (WLR) in the first 3–6 months and overall WLR were categorized as Fast WLR (≥1 kg/wk), Recommended WLR (0.5 to 0.9 kg/wk), or Slow WLR (<0.5 kg/wk).

**Results:**

On average, patients attained a 6.6 ± 7.3 kg (5.8 ± 5.7%) WL over 12.8 ± 13.1 months. Prior to adjusting for covariates, patients with Fast WLR (−24.7 ± 13.4 kg) at 3–6 months had a greater overall WL as compared to those with Recommended WLR (−13.3 ± 8.7 kg) and Slow WLR (−5.0 ± 5.4 kg). Fast WLR also had greater improvements in the overall waist circumference and blood pressure than patients with Slow or Recommended WLR. However, after adjustment for absolute WL, Early and overall Recommended and Fast WLR did not differ in the changes in any of the health markers (*P* > 0.05). Conversely, the absolute WL sustained is significantly associated with changes in metabolic health independent of WLR (*P* < 0.001). Similar results were observed with WLR over the entire treatment period.

**Conclusions:**

Faster rates of WL are associated with a greater absolute WL and larger improvements in waist circumference and blood pressure. However, after adjusting for the larger absolute WL sustained, early and overall faster WLR do not appear to have advantages for improving metabolic health markers. Thus, the absolute WL attained may be the most important factor for improving metabolic health.

## 1. Introduction

Current weight management guidelines emphasize the importance of attaining a 5% weight loss (WL) to achieve health benefits [1, 2]. However, it is unclear whether the rate of WL influences health beyond the absolute weight change attained.

Interestingly, the recommended 1-2 lb/wk (∼0.5 to −1 kg/wk) WLR (Rec WLR) in the 1998 guidelines [3] was based on reducing the risk for gallstone formation [3–5]. Faster WL has long been documented to be associated with larger WL overall [6, 7], but has historically thought to be associated with worse or no better long-term weight management [6, 8]. However, a review by Astrup and Rössner suggests that individuals with greater initial WLR may also be associated with better long-term weight loss maintenance when long-term weight management care is given [6]. Given that obesity is now viewed as a chronic disease, long-term care should be an expectation for obesity management, and weight maintenance programs of over a year are currently recommended by the guidelines [9]. To date, most studies examining WLR have examined the impact on body composition [10, 11] or resting metabolic rate [10–12] and report no significant differences, though they may have been underpowered. We are aware of only three studies other than our preliminary analysis that has examined the influence of WLR on CVD risk factors [13–15]. These intervention studies designed the WL to be similar between groups and did not observe any differences in BP or lipid changes between fast and slow WL but may have been underpowered. We have previously shown that faster WL is associated with greater WL and reductions in BP, but after adjustment for overall WL, there were no significant differences in BP reduction between fast and slow WL [16]. However, these studies did not directly examine whether WLR faster than the commonly recommended 1-2 lb/wk results in differential effects on health independent of the absolute WL and thus warrants further investigation.

Thus, the objective of the current study is to determine whether the rate of WL is associated with changes in health factors independent of the absolute WL.

## 2. Materials and Methods

### 2.1. Study Population

The study population included 11,283 patients who attended the Wharton Medical Clinic (WMC) Weight Management Program between July 2008 and July 2017. Data up to 5 years of treatment were obtained from electronic medical files. To ensure a fairer comparison among WLR groups, patients were included only if they attended the clinic for at least 3 months and lost at least 0.1 kg of weight (i.e., those who lost weight) and had repeated metabolic variable measurements. Patients who had bariatric surgery were excluded from the analysis (*n*=25). All participants gave their written consent for the use of their medical data for research purposes and were informed that their decision to participate would not influence their medical treatment at WMC. Participants did not receive any form of stipends. All methods were approved by the York University Research Ethics Board (Ethics Certificate #: 2009–117, 2013–123, e2017–166).

As described previously [16–18], WMC is a physician referral-based clinic designed to educate and enable patients to implement strategies to manage their weight and improve their health. Patients attend the clinic on a monthly basis or as needed to have individual meetings with bariatric educators (with a university degree in nutrition) to discuss personalized weight management strategies, dietary plans, and physical activity options. Patients meet with physicians to discuss medication options, interest in bariatric surgery, and any obesity-related comorbidities. If indicated, patients are referred for additional tests, to other medical professionals, or for bariatric surgery. Patients can attend the program for as long as they wish and are able to return to the clinic after long absences. The clinic operates within the Ontario Health Insurance Plan, and all services are provided at no charge to the patient.

Patients undergo a standard battery of clinical tests including waist circumference, blood pressure (BP), fasting glucose, triglycerides (TG), and high-density lipoprotein (HDL) assessed using standard clinical methods. Participants were included if they had change data for at least one health measure (*n* = 1,697 to  11,223); thus, the sample size for the metabolic data analyses varied depending on the availability of data.

Body weight (BW) was measured by staff at each patient visit on a calibrated MedWeight, MS-2510 Digital High Capacity Platform Scales (Itin Scale Co, Inc., NY). Weight change was calculated as final observed BW-initial BW. For each health measure, the final weight that most closely corresponded to the health measure timeframe was used. The rate of weight loss was calculated as the weight change/treatment time during the first 3–6 months and over the entire treatment period that exceed, met, or was slower than the 1-2 lb/wk (∼0.45–0.91 kg/wk) recommendation: Fast WLR (≥0.91 kg/wk), Rec WLR (0.45 to 0.90 kg/wk), or Slow WLR (<0.45 kg/wk).

### 2.2. Statistical Analyses

Patient characteristics and baseline metabolic variables were presented as means ± SD stratified by early WLR. Group differences in participant characteristics were assessed using an analysis of variance for continuous variables and chi-square tests for categorical variables. The independent associations of overall and early WL and the rate of WL (continuous and categorical with Rec WLR as the reference group) on changes in health markers were adjusted for age, sex, baseline values, relevant medication (yes/no), and use of weight loss medications (yes/no). Results were similar when only baseline metabolic value and absolute WL were adjusted for, and thus, only the final model is presented. Bonferroni post hoc tests were used. All statistical analyses were conducted using SAS 9.4 (SAS Institute, Cary, NC, USA). Statistical significance was established at *P* < 0.05.

## 3. Results

Participant characteristics stratified by early WLR is shown in Table 1. In general, those with early Fast WLR were younger and had a higher BMI than those with Slow WLR or Rec WLR. Patients had a greater overall WL in Fast WLR (−24.7 ± 13.4 kg) at 3–6 months as compared with those with Rec WLR (−13.3 ± 8.7 kg) and Slow WLR (−5.0 ± 5.4 kg). Furthermore, early Fast WLR had greater overall improvements in waist circumference (−15.2 versus −9.5 or 4.2 cm) and blood pressure (SBP: −9 versus −7 or −3 mmHg; DBP: −6 versus −4 or −2 mmHg) than patients with early Slow WLR or Rec WLR (*P* < 0.05 for all). These differences remained true with adjustment for age, sex, baseline body weight, treatment time, and WL medication use (results not shown).

**Figure 1:**
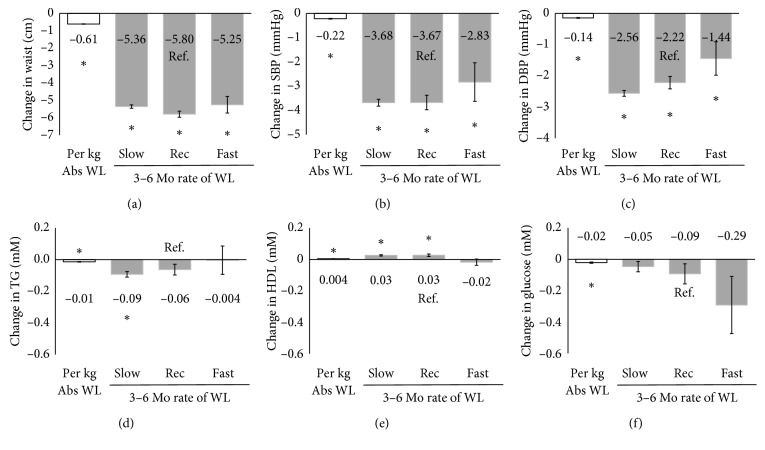
Absolute weight loss, but not early rate of WL, during the first 3–6 months is associated with changes in health markers with adjustment for absolute weight loss. ^*∗*^Significantly associated with changes in health markers adjusting for rate of WL, age, sex, treatment time, weight loss medication, baseline value, and relevant medications (*P* < 0.0001). No significant difference between WLR groups (*P* > 0.05).

In a model adjusting for absolute WL, patients with early Slow WLR, Rec WLR, and Fast WLR had similar improvements in waist and BP (*P* < 0.05), but modest to no changes in the lipids or glucose (Figure 1). After adjusting for absolute WL, WLR groups no longer differed in the changes in any of the health markers or waist circumference over the intervention (*P* > 0.05), whereas absolute WL remained independently associated with changes in all of the health markers (*P* < 0.0001). When early WLR during the first 3 to 6 months was examined as a continuous variable, the WLR was associated with greater reductions in glucose independent of absolute weight loss (*β* = −0.34 mM/kg/wk, *P*=0.009), but there was no association between early WLR and the other metabolic risk factors (*P* > 0.05).

**Figure 2:**
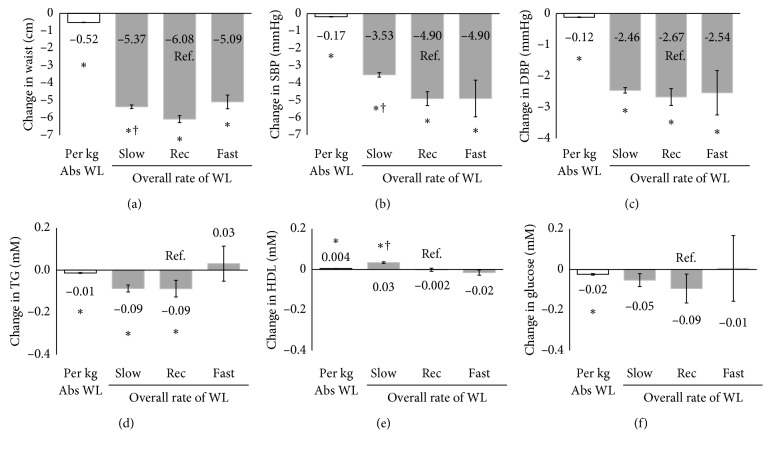
Independent associations between the overall rate of WL and absolute weight loss with changes in health markers during the entire treatment period. ^*∗*^Significantly associated with changes in health markers adjusting for rate of WL, age, sex, treatment time, weight loss medication, baseline value, and relevant medications (*P* < 0.0001). ^†^Significant difference from Slow WLR group (*P* < 0.05). Rec WLR and Fast WLR did not differ (*P* > 0.05).

Similarly, when examining the rate of WL independent of absolute WL over the entire intervention, the overall Rec WLR and Fast WLR did not differ in changes in any of the health markers (*P* > 0.05, Figure 2). The overall Slow WLR had significantly smaller reductions in waist circumference and SBP as compared with Rec WLR (*P* < 0.05). Conversely, the overall Slow WLR had significantly better improvements in HDL as compared with Rec WLR and Fast WLR (*P* < 0.05). As with early WLR, absolute WL remained significantly associated with all health changes independent of the overall WLR (Figure 2, *P* < 0.0001). When the WLR over the entire intervention was examined as a continuous variable, the WLR was associated with greater improvements in SBP (*β* = −2.57 mmHg/kg/wk, *P* < 0.0001), but smaller improvements in HDL (*β* = −0.06 mM/kg/wk, *P* < 0.0001).

## 4. Discussion

Our study expands on traditional examinations of WL, by illustrating that the superior improvements in obesity reduction and health improvements associated with faster WL as compared to the commonly recommended 1-2 lb/wk are abolished after adjusting for greater WL. Conversely, absolute weight loss is significantly associated with improvements in all health risk factors. Thus, obesity interventions should focus on long-term weight loss maintenance.

Current weight management guidelines generally recommend a WLR of 1-2 lb/wk (0.45–0.91 kg/wk) with a 5% WL target overall to achieve health benefits [2, 3, 8]. However, this recommended WLR is for reducing the risk for gallstone formation [4] and was not created based on associations with obesity or cardiometabolic health outcomes. In accordance with previous observations [7, 16], faster WLR is associated with greater WL overall as compared to slower WLR. We extend these findings to show that there is also a greater reduction in waist circumference with faster WLR. This is in contrast with previous smaller studies that report similar reductions in waist [14] and body fat [11, 14, 19] with fast versus slow WLR. However, some of these studies were also designed to be similar in WL attained. After controlling for differences in WL, we also observe that there were similar reductions in waist by WLR. In the model with the overall WLR, there was a small statistically significant smaller reduction in waist (0.71 cm) with slower WLR than for weight losses of 1-2 lb/wk that is within the range for measurement error, particularly for populations with severe obesity [20]. Thus, it appears that the body compositional changes with fast and slow WLR may be similar after accounting for differences in absolute WL.

Whether the WLR results in differential effects on health independent of the absolute WL is unclear. Three studies in smaller cohorts report that there are no differences in health changes between fast and slow WL [13–15]. Using a substantively larger sample, we demonstrate that WLR faster than the recommended 1-2 lb/wk is associated with greater improvements in BP [16]. However, after adjustment for the differences in absolute WL, there were minimal differences in the metabolic risk factor changes, with the differences tending to be worse in the Fast WLR as compared with the Rec WLR group. When weight loss rate was examined as a continuous variable, we observe significantly better reductions in glucose and SBP with faster WLR. Together, these results indicate that the benefits of faster WLR on health occur at rates slower than the recommended 1-2 lb/wk WLR, with no additional benefits for WLR faster than the recommended rate. Conversely, slower WLR was associated with better improvements in HDL than the recommended 1-2 lb/wk. However, these differences are likely not of a magnitude that would be clinically relevant. Many of these differences are below the measurement accuracy for these tests and are well within the day-to-day variations expected. Thus, our results would suggest there are minimal, if any, cardiometabolic benefits or consequences associated with rapid WL after adjusting for differences in absolute weight loss. Taken together, we conclude that absolute WL should be the focus for improving health, and faster WLR may be an avenue that patients may be able to attain greater weight loss and long-term weight maintenance [6], but at the small increased risk for gallstone formation [4]. However, if the same WL is attainable through the recommended 1-2 lb/wk WLR, then that would be the most optimal choice for weight and health outcomes.

Strengths and limitations of the current study warrant mention. Our study sample consisted of patients from a publicly funded weight management clinic that was predominately middle-aged women, and the applicability of these findings to other demographics is unclear. The individuals included in this analysis were generally at the clinic for less than 2 years, and thus, we are unsure of the long-term sustainability of their WL, weight regain, or health changes. Because of the nature of care provided at the weight management clinic, reporting of gall stones is inconsistent, as care would be provided at the hospital and follow-up treatment by their primary care physician. Nevertheless, the risk of gallstones is well documented and important to consider. Furthermore, as only two time points were used, we cannot speak to the influence of weight loss patterns on the health changes; however, it is suggested that the weight regain after fast and slow WL is similar [7, 14, 19]. Finally, this was mainly individualized clinical weight management care that predominately focuses on dietary interventions, and we cannot say for certain what specific intervention components or external factors lead to the weight or health changes observed. However, the use of weight loss medications was adjusted for in the analyses.

In summary, we demonstrate that patients with rates of WL faster than the commonly recommended 1-2 lb/wk (0.45–0.91 kg/wk) tended to have greater obesity reduction and superior health improvements than slower WL. However, the superior improvements in health associated with faster WL are abolished after adjusting for absolute WL. Faster WLRs either early in the intervention or over the entire treatment were associated with similar changes in metabolic health at the commonly recommended rate in patients attending a publicly funded clinical weight management program. Thus, future WL interventions aimed at improving metabolic health should focus on the absolute weight loss attained and long-term weight management.

## Figures and Tables

**Table 1:** Subject characteristics.

Variable	Slow WLR		Rec WLR		Fast WLR	
	N	Mean (SD)	N	Mean (SD)	N	Mean (SD)
Age (y)	9179	52.6 (13.1)	1851	52.4 (12.7)	251	50.7 (11.5)^*∗*^
Sex (%male)		26.1%		33.9%^*∗*^		60.1%^*∗*^^†^
Treatment time (mo)		12.2 (12.6)		15.1 (14.7)^*∗*^		15.0 (15.5)^*∗*^
BMI (kg/m^2^)		39.3 (7.2)		42.7 (8.2)^*∗*^		47.8 (10.8)^*∗*^^†^
Weight (kg)		109.3 (23.8)		121.9 (26.9)^*∗*^		142.4 (28.7)^*∗*^^†^
Δ weight (kg)		−5.0 (5.4)		−13.3 (8.7)^*∗*^		−24.7 (13.4)^*∗*^^†^
Overall WLR (kg/wk)		−0.13 (0.11)		−0.37 (0.24)^*∗*^		−0.73 (0.51)^*∗*^^†^
Early WLR (kg/wk)		−0.19 (0.14)		−0.61 (0.11)^*∗*^		−0.93 (0.46)^*∗*^^†^
SBP (mmHg)	9109	128 (13)	1840	132 (14)^*∗*^	249	135 (15)^*∗*^^†^
Δ SBP (mmHg)		−3 (13)		−7 (14)^*∗*^		−9 (15)^*∗*^^†^
DBP (mmHg)	9073	78 (8)	1836	79 (8)^*∗*^	247	82 (9)^*∗*^^†^
Δ DBP (mmHg)		−2 (9)		−4 (9)^*∗*^		−6 (10)^*∗*^^†^
Glucose (mM)	1344	6.0 (1.5)	305	5.8 (1.3)	41	6.0 (1.2)
Δ glucose (mM)		−0.1 (0.9)		−0.2 (0.9)^*∗*^		−0.5 (0.9)^*∗*^
Triglyceride (mM)	1524	1.6 (0.8)	346	1.5 (0.8)	46	1.4 (0.7)
Δ triglyceride (mM)		−0.1 (0.6)		−0.1 (0.6)		−0.2 (0.5)
HDL (mM)	2253	1.26 (0.33)	469	1.20 (0.32)^*∗*^	67	1.10 (0.25)^*∗*^
Δ HDL (mM)		0.02 (0.15)		0.06 (0.17)^*∗*^		0.05 (0.15)
Waist (cm)	4868	120.1 (15.5)	1360	126.5 (16.8)^*∗*^	172	140.2 (16.1)^*∗*^^†^
Δ waist (cm)		−4.2 (6.7)		−9.5 (8.3)^*∗*^		−15.2 (11.9)^*∗*^^†^
Weight loss med use (%)	9179	14.6%	1851	17.0%	253	18.1%

Slow WLR (<0.45 kg/wk), Rec WLR (0.45 to 0.9 kg/wk), or Fast WLR (≥0.91 kg/wk) during the initial 3–6 month. ^*∗*^Different from Slow WLR (*P* < 0.05). ^†^Different from Rec WLR (*P* < 0.05).

## Data Availability

The datasets generated and analysed during the current study are not publicly available due to privacy laws associated with medical data, but are available with a data sharing agreement as approved by the relevant institutional ethics committee and the health information custodian (Sean Wharton).

## Additional Points

What is known? (i) Faster weight loss is associated with greater overall weight loss than slower weight loss. (ii) Weight loss of faster than 2 lb/wk (∼1 kg/wk) is associated with increased risk for gallstone formation. What does this study add? (i) Faster weight loss is associated with greater weight loss and health improvements than slow weight loss. (ii) Fast and slow weight losses are associated with similar improvements in metabolic health after adjusting for the absolute weight loss attained.
